# *Biomphalaria glabrata *transcriptome: cDNA microarray profiling identifies resistant- and susceptible-specific gene expression in haemocytes from snail strains exposed to *Schistosoma mansoni*

**DOI:** 10.1186/1471-2164-9-634

**Published:** 2008-12-29

**Authors:** Anne E Lockyer, Jenny Spinks, Richard A Kane, Karl F Hoffmann, Jennifer M Fitzpatrick, David Rollinson, Leslie R Noble, Catherine S Jones

**Affiliations:** 1 Wolfson Wellcome Biomedical Laboratories, Department of Zoology, The Natural History Museum, London, SW7 5BD, UK; 2 Department of Pathology, Cambridge University, Tennis Court Road, Cambridge, UK; 3 Institute of Biological and Environmental Sciences, School of Biological Sciences, Zoology Building, Tillydrone Avenue, University of Aberdeen, Aberdeen, AB24 2TZ, UK; 4 Institute of Biological Sciences, Edward Llwyd Building, University of Wales, Aberystwyth, Ceredigion, SY23 3DA, UK; 5 Department of Medicine, University of Southampton, Bassett Crescent East, Southampton, Hampshire SO16 7PX, UK

## Abstract

**Background:**

*Biomphalaria glabrata *is an intermediate snail host for *Schistosoma mansoni*, one of the important schistosomes infecting man. *B. glabrata/S. mansoni *provides a useful model system for investigating the intimate interactions between host and parasite. Examining differential gene expression between *S. mansoni*-exposed schistosome-resistant and susceptible snail lines will identify genes and pathways that may be involved in snail defences.

**Results:**

We have developed a 2053 element cDNA microarray for *B. glabrata *containing clones from ORESTES (Open Reading frame ESTs) libraries, suppression subtractive hybridization (SSH) libraries and clones identified in previous expression studies. Snail haemocyte RNA, extracted from parasite-challenged resistant and susceptible snails, 2 to 24 h post-exposure to *S. mansoni*, was hybridized to the custom made cDNA microarray and 98 differentially expressed genes or gene clusters were identified, 94 resistant-associated and 4 susceptible-associated. Quantitative PCR analysis verified the cDNA microarray results for representative transcripts. Differentially expressed genes were annotated and clustered using gene ontology (GO) terminology and Kyoto Encyclopaedia of Genes and Genomes (KEGG) pathway analysis. 61% of the identified differentially expressed genes have no known function including the 4 susceptible strain-specific transcripts. Resistant strain-specific expression of genes implicated in innate immunity of invertebrates was identified, including hydrolytic enzymes such as cathepsin L, a cysteine proteinase involved in lysis of phagocytosed particles; metabolic enzymes such as ornithine decarboxylase, the rate-limiting enzyme in the production of polyamines, important in inflammation and infection processes, as well as scavenging damaging free radicals produced during production of reactive oxygen species; stress response genes such as HSP70; proteins involved in signalling, such as importin 7 and copine 1, cytoplasmic intermediate filament (IF) protein and transcription enzymes such as elongation factor 1α and EF-2.

**Conclusion:**

Production of the first cDNA microarray for profiling gene expression in *B. glabrata *provides a foundation for expanding our understanding of pathways and genes involved in the snail internal defence system (IDS). We demonstrate resistant strain-specific expression of genes potentially associated with the snail IDS, ranging from signalling and inflammation responses through to lysis of proteinacous products (encapsulated sporocysts or phagocytosed parasite components) and processing/degradation of these targeted products by ubiquitination.

## Background

Schistosomiasis, the most widespread trematode infection, is estimated to infect around 200 million people in 75 countries of the developing world, leading to chronic debilitating disease and up to 200 000 deaths per year [1]. The freshwater snail *Biomphalaria glabrata *is an intermediate host for *Schistosoma mansoni*, a digenean parasite that causes human intestinal schistosomiasis. This medically relevant gastropod is a member of one of the largest invertebrate phyla, the Mollusca, which are lophotrochozoans, a lineage of animal evolution distinct from ecdysoans, represented by present model invertebrates such as *Caenorhabditis *and *Drosophila*. Many of the genomic studies in molluscs have focussed on bivalves owing to the importance of these organisms in aquaculture and fisheries and to their role in marine environmental science [2,3], while in gastropods, expressed sequence tag (EST) studies have been carried out in *Lymnaea stagnalis *[4] and *B. glabrata *[5-7]. Characterizing genes and biochemical pathways central to immunity and defence in gastropods is predicted to reveal innovative data, significant due to the medical and economic importance of intermediate host snails in parasite transmission, and *B. glabrata *is poised to become the next invertebrate model organism. Indeed, advances towards the ultimate goal of obtaining the *B. glabrata *genome sequence [8] include the complete mitochondrial genome [9], the development of a BAC library for genome sequencing [10] and complementary gene discovery projects [5-7]. Interactions between snails and schistosomes are complex and there exists an urgent need to elucidate pathways involved in snail-parasite relationships as well as to identify those factors involved in the intricate balance between the snail internal defence system (IDS) and trematode infectivity mechanisms that determine the success or failure of an infection [for a review see [11]].

Molluscs appear to lack an adaptive immune system like that found in vertebrates and, instead, are considered to use various innate mechanisms involving cell-mediated and humoral reactions (non-cellular factors in plasma/serum or haemolymph) that interact to recognize and eliminate invading pathogens or parasites in incompatible or resistant snails [for reviews see [12-14]]. However, a diverse family of fibrinogen related proteins (FREPs) containing immunoglobulin-like domains has been discovered in *B. glabrata *and may play a role in snail defence [15]. Circulating haemocytes (macrophage-like defence cells) in the snail haemolymph are known to aggregate in response to trauma, phagocytose small particles (bacteria, and fungi) and encapsulate larger ones, such as parasites. Final killing is effected by haemocyte-mediated cytotoxicity mechanisms involving non-oxidative and oxidative pathways, including lysosomal enzymes and reactive oxygen/nitrogen intermediates [16,17]. Certain alleles of cytosolic copper/zinc superoxide dismutase (SOD1) have been associated with resistance [18,19] also suggesting these processes are important in the snail IDS.

Compatible snail-trematode infections may reflect the parasite's capacity to avoid or interfere with the innate response of the snail. The term 'resistant' can be applied to those individuals within a single snail species that are able to evade infection by a species or strain of schistosome that is normally capable of parasitizing that species of snail. Identification of specific molecules mediating the defensive events in snail intermediate hosts, in particular those differentially expressed in resistant/incompatible snails, is expected to reveal much about the pathways and processes involved. Previous studies of differential gene expression in resistant and susceptible snail lines have used a number of different techniques including differential display [20-25] and suppression subtractive hybridization (SSH) [26,27] to identify in each case, some differentially expressed genes. The application of cDNA microarray technology allows large-scale analysis of differential gene expression, using large numbers of sequenced cDNA clones, which will enable a more detailed examination of the *B. glabrata *transcriptome. This paper describes the construction of the first *B. glabrata *microarray using previously sequenced ORESTES clones [6] as well as new sequences from SSH libraries enriched for differentially expressed genes, and demonstrates its use in detecting differentially expressed transcripts in the haemocytes of parasite-challenged resistant and susceptible snail lines, with the aim of identifying strain-specific transcripts potentially involved in snail internal defence.

## Results

### Sequence analysis of SSH and ORESTES clones for microarray fabrication

To initiate large scale analysis of differential gene expression in *B. glabrata*, a cDNA microarray was constructed from 1062 previously sequenced PCR-amplified ORESTES clones [6] and, in addition to these, 980 new sequenced clones from existing SSH libraries [27]. It also contains transcripts derived from our previous expression studies [20-22]. The ORESTES clones were from the first 27 Bg ORESTES libraries [6], available at the time of printing and were derived from 4 different tissues (ovotestis, headfoot, haemopoietic organ and haemocytes), from the two snail strains used in this experiment, NHM3017 (resistant) and NHM1742 (susceptible). All the available EST clones were printed, including those from tissues other than haemocytes, since it could not be ascertained *a priori *that none of these would be involved in snail defence, or that transcripts originally derived from tissues other than haemocytes would definitely not be expressed in the haemocytes. The analyzed ESTs were screened to remove duplicates within each library. In addition to the clones derived from the ORESTES libraries, 8 haemocyte and haemopoietic organ (the organ responsible for producing the haemocytes) SSH libraries (described in [27]) were available for sequencing and clone selection. Two to three 96 well plates of clones were sequenced from each library. A total of 1728 sequences were obtained (Table 1), of which just over 2% (n = 40) had been previously sequenced from *B. glabrata*. Over 35% had no known identity (n = 606), either having no match in publicly available sequence databases, or matching a protein or other EST that had no known identity. After removal of duplicate, short (< 80 bp) and ribosomal sequences, 980 were available for printing on the microarray.

**Table 1:** Blast results summary.

	All sequences		Non-redundant sequences	
Category	N° sequences	% sequences	N° sequences	% sequences

Protein	281	16.26	275	28.06
Mitochondrial protein	91	5.27	63	6.43
Ribosomal protein	37	2.14	36	3.67
*Biomphalaria *protein	10	0.58	7	0.71
*Biomphalaria *fragment	30	1.74	20	2.04
Ribosomal	315	18.23	2	0.20
Unknown (no BLAST match)	525	30.38	497	50.71
Unknown EST	46	2.66	40	4.08
Unknown protein	35	2.03	33	3.37
Other	358	20.72	7	0.71

Total	1728	100.00	980	100.00

Analysis of the types of sequences obtained from the *B. glabrata *SSH libraries identified with Blast searches of GenBank.

**Figure 1:**
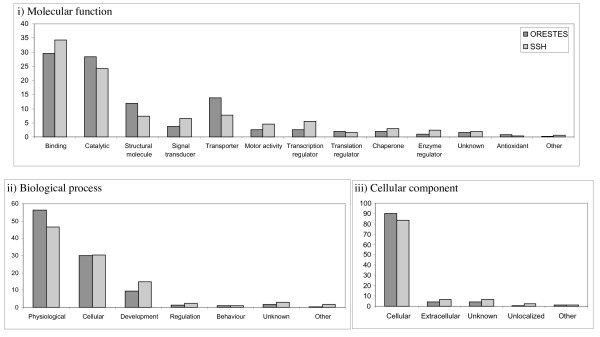
**Gene Ontology assignments to compare the percentage of clones from ORESTES and SSH in each category for i) molecular function; ii) biological process and iii) cellular component**.

The available ESTs from SSH and ORESTES (n= 2042) were compared to GenBank and where possible gene ontology was assigned. The assignments in all three categories, molecular function, biological process and cellular component show no obvious differences in the sequence types obtained from the two different methods (Fig. 1). An analysis of metabolic pathways using the Kyoto Encyclopaedia of Genes and Genomes (KEGG; see Additional file 1) showed that, generally, genes from similar pathways were identified from both ORESTES and SSH, for instance, genes implicated in the immune system showed similar numbers in each pathway from both techniques. However, there were many more genes involved in oxidative phosphorylation sequenced from SSH (76 clones, representing 27 different enzymes) when compared to ORESTES (6 clones, representing 6 different enzymes) and the same was seen with the numbers of ribosomal proteins from SSH (37 clones, representing 32 different enzymes) compared to ORESTES (7 clones, representing 7 different enzymes).

### Expression profiling by microarray analysis

The microarray was used for a direct comparison of mRNA from haemocytes of parasite-exposed resistant and susceptible snails. Haemocytes sampled over the first 24 h post parasite exposure were compared to investigate differences between snail lines after parasite exposure during the period when the haemocytes are thought to respond and encapsulate the parasite in resistant snails [28]. This approach was designed to identify large and significant differences in gene expression between the two snail lines, although small, transient RNA expression changes might not be identified. Since very small amounts of RNA were available from the haemocytes, independent SMART amplifications [29] and labelling reactions were carried out and four technical replicate hybridizations performed. SMART amplification has been shown to have a higher true-positive rate than global amplification, but has a lower absolute discovery rate, and a systematic compression of observed expression ratio [30].

Analysis of duplicate spots on each array showed good correlation of normalized mean pixel intensity ratios (correlation coefficients 0.9577–0.9889) demonstrating that the hybridizations were consistent within each array (results not shown). The data were screened to remove weak signals below the level of background hybridization in both channels based on negative control vector levels of hybridization. Comparisons were made between technical replicate arrays, of mean (from duplicate features) normalized mean pixel intensity ratios obtained for each clone, using the data that passed the filtering and background (vector) threshold (see Additional file 2). Correlation coefficients for each array comparison were high, ranging from 0.8452 to 0.9604, showing good agreement in gene expression values between array hybridizations, suggesting that SMART amplification of the cDNA was not affecting representation of transcripts. The amplified cDNA demonstrated a good degree of hybridization reproducibility and a low level of variation between technical replicates, giving confidence to the assignment of differentially expressed strain-associated transcripts.

**Figure 2:**
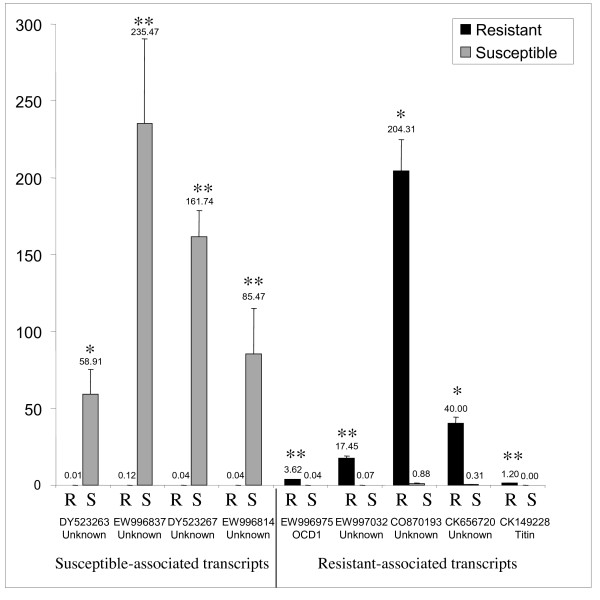
**Histogram showing relative (normalized against actin) expression from qPCR for 9 genes in unamplified cDNA from schistosome-exposed resistant and susceptible snails**. The P values from a students t-test demonstrated * < 0.05 or ** < 0.01 significance.

To identify genes showing differential expression between the resistant and susceptible snail haemocytes, 99% confidence intervals were calculated based on the remaining data, but excluding data from SSH clones. These were not included in calculating significance intervals, since the 99% confidence intervals are based on the assumption that the clones are normally distributed; however since the SSH clones were enriched for differential expression this assumption is not true for these clones. Clones that fell outside the 99% confidence interval in 3 or more of the 4 replicates were designated differentially expressed (Table 2). A total of 110 differentially expressed transcripts were identified, 105 were from the resistant haemocytes and only 5 were from the susceptible haemocytes. All of these identified genes showed an average (across the 4 replicates) of between 3 and 5.3 fold difference in expression between resistance and susceptible snails (Table 2). Nine candidates, 4 susceptible-associated transcripts and 5 resistant-associated transcripts, were selected for confirmation of differential expression with qPCR using unamplified cDNA (Fig. 2). In each case the qPCR result confirmed the result found from the microarray. The difference in expression of all of these genes was much greater than that indicated in the array; in many cases the transcript was barely detectable in the strain which showed less expression from the microarray. This confirms the suggestion that SMART amplification compresses the observed expression ratio and may also suggest that the fold difference in gene expression observed from the microarray underestimates the true difference in expression of transcripts.

**Table 2:** Differentially expressed genes.

dbEST Ac No	Mean	Source^a^	BlastX match^b^	Organism	Acc No	Expect
EW996975*	-5.261	SSH	Ornithine decarboxylase 1	*Danio rerio*	NP_571876.1	3E-16
EW997424	-5.124	SSH	Unknown			
EW997539	-4.996	SSH	Unknown			
CK656700	-4.845	ORESTES	Multidrug resistance transporter-like protein	*Pseudopleuronectes americanus*	AAL15148.1	2E-34
CO870193*	-4.816	ORESTES	Unknown			
CK149228*	-4.806	ORESTES	Titin	*Homo sapiens*	CAA49245.1	3E-17
EW997032*	-4.801	SSH	Unknown			
CK656739	-4.772	ORESTES	Unknown^2^			
EW997027	-4.698	SSH	Unknown			
EW997530	-4.698	SSH	Unknown			
CK656724	-4.696	ORESTES	Unknown			
EW997444	-4.643	SSH	MBCTL1	*Monosiga brevicollis*	AAP78680.1	5E-04
CO870221	-4.597	ORESTES	Unknown^1^			
CK656720*	-4.594	ORESTES	Unknown			
EW997542	-4.544	SSH	Unknown			
CK656728	-4.539	ORESTES	Crooked neck-like 1 protein	*Mus musculus*	NP_080096.1	1E-105
EW997099	-4.488	SSH	Unknown^9^			
EW996832	-4.472	SSH	NADH ubiquinone oxidoreductase	*Caenorhabditiselegans*	NP_496376.1	1E-30
EW997050	-4.459	SSH	Bystin	*H. sapiens*	AAH50645.1	2E-34
EW996838	-4.417	SSH	Unknown			
EW996831	-4.407	SSH	Unknown			
EW997370	-4.382	SSH	Unknown			
CO870223	-4.361	ORESTES	Unknown^1^			
EW997028	-4.316	SSH	Unknown			
EW997117	-4.306	SSH	Unknown			
EW997432	-4.303	SSH	Unknown			
CK656726	-4.299	ORESTES	Cytoplasmic intermediate filament protein	*Ascaris lumbricoides*	CAA60047.1	3E-27
EW997187	-4.287	SSH	Unknown			
EW997456	-4.264	SSH	Unknown			
EW996835	-4.248	SSH	Unknown			
EW997566	-4.247	SSH	Unknown			
CK656741	-4.225	ORESTES	Ubiquitin	*Suberites domuncula*	CAA76578.1	9E-26
EW996808	-4.224	SSH	NHL domain containing protein	*C. elegans*	NP_499028.1	4E-10
EW996834	-4.201	SSH	Unknown			
CO870200	-4.195	ORESTES	Unknown^1^			
EW997451	-4.193	SSH	Unknown			
EW997446	-4.158	SSH	Importin 7; RAN-binding protein 7	*D. rerio*	NP_006382.1	9E-40
EW997144	-4.122	SSH	Unknown			
EW996812	-4.120	SSH	Unknown			
EW997049	-4.109	SSH	Unknown			
EW997528	-4.103	SSH	Myosin light chain kinase smooth	*Bos taurus*	Q28824	7E-09
EW997428	-4.094	SSH	Hypothetical protein	*Deinococcus radiodurans*	NP_294693.1	2E-15
EW997118	-4.068	SSH	Unknown^8^			
CK656734	-4.056	ORESTES	Unknown^1^			
CK149216	-4.053	ORESTES	Unknown			
EW997092	-4.050	SSH	Unknown^8^			
EW997556	-4.035	SSH	Unknown			
EW997531	-4.021	SSH	Unknown			
CK656703	-3.992	ORESTES	Sqstm1 protein	*M. musculus*	AAH06019.1	0.0003
EW997407	-3.976	SSH	Unknown			
EW997035	-3.971	SSH	Unknown			
EW997077	-3.961	SSH	Unknown			
CK656698	-3.954	ORESTES	Unknown			
EW997078	-3.932	SSH	Unknown		.	.
CK656711	-3.932	ORESTES	ADP/ATP carrier^2^	*Neocallimastix patriciarum*	AAL79525.1	1E-30
EW997555	-3.920	SSH	Elongation factor-2^7^	*Rattus norvegicus*	CAC81931.1	3E-32
EW996976	-3.915	SSH	Ornithine decarboxylase	*C. elegans*	NP_504752.1	2E-25
EW997411	-3.892	SSH	Lactate/malate dehydrogenase	*C. elegans*	NP_872154.1	5E-09
EW997017	-3.890	SSH	Fumarylacetoacetate hydrolase-related protein	*A. thaliana*	NP_172669.2	2E-37
EW997501	-3.881	SSH	Unknown			
EW996972	-3.875	SSH	Unknown^6^			
EW997045	-3.864	SSH	ATF6	*H. sapiens*	BAA34722.1	0.0004
CK656733	-3.830	ORESTES	Ubiquitin-conjugating enzyme E2D 2	*D. rerio*	AAH48896.1	2E-18
EW997087	-3.827	SSH	Unknown			
EW996855	-3.824	SSH	Unknown			
EW997194	-3.813	SSH	Unknown^9^			
CK656713	-3.797	ORESTES	Unknown			
CO870211	-3.796	ORESTES	Unknown			
EW997015	-3.785	SSH	Unknown			
EW997129	-3.758	SSH	Unknown			
EW997449	-3.757	SSH	Unknown^5^			
EW997533	-3.754	SSH	Unknown			
EW997418	-3.740	SSH	Hypothetical protein	*Aspergillus nidulans*	EAA64844.1	7E-11
EW996827	-3.736	SSH	Serine 2 transmembrane protease	*M. musculus*	NP_056590.1	4E-25
CK656737	-3.734	ORESTES	70 kDa heat shock protein^3^	*Artemia franciscana*	AAL27404.1	5E-59
CK656706	-3.723	ORESTES	M-phase phosphoprotein 1	*H. sapiens*	NP_057279.1	4E-4
CO870188	-3.720	ORESTES	Cathepsin L-like protease precursor	*A. franciscana*	AAD39513.1	3E-5
EW997548	-3.683	SSH	Gnat-family acetyltransferase	*Bordetella parapertussis*	NP_883664.1	6E-29
EW997412	-3.677	SSH	Cytochrome b	*Macaca tonkeana*	AAK26394.1	7E-51
EW997170	-3.669	SSH	Unknown^6^			
EW996822	-3.593	SSH	Unknown			
EW997067	-3.569	SSH	Elongation factor 2^7^	*Entamoeba histolytica*	Q06193	8E-6
EW997053	-3.558	SSH	Elongation factor 1-alpha	*Coccidioides immitis*	Q96WZ1	1E-12
CK656707	-3.550	ORESTES	Heat shock protein 70^3^	*Ambystoma mexicanum*	AAK31583.1	1E-67
CK656701	-3.541	ORESTES	Unknown			
EW997447	-3.511	SSH	Unknown			
EW997408	-3.502	SSH	Unknown			
EW996986	-3.501	SSH	Ribosomal protein Rps2	*Cricetulus griseus*	AAQ94085.1	6E-08
EW997107	-3.483	SSH	Unknown			
EW997105	-3.478	SSH	Streptavidin V2 precursor	*Streptomyces violaceus*	Q53533	7E-5
EW997127	-3.472	SSH	Cytochrome oxidase subunit I	*Platynectes decempunctatus*	AAN11289.1	1E-40
EW996813	-3.472	SSH	Unknown			
EW996825	-3.462	SSH	Unknown^5^			
EW996824	-3.461	SSH	Adenosylhomocysteinase 3	*R. norvegicus*	XP_231564.1	5E-43
CO870190	-3.460	ORESTES	Glutamyl-prolyl-tRNA synthetase	*D. melanogaster*	NP_524471.2	4E-56
EW997004	-3.458	SSH	Unknown			
EW997180	-3.435	SSH	Unknown^1^			
EW997569	-3.430	SSH	Oligomycin sensitivity conferring protein	*D. melanogaster*	CAA67980.1	1E-23
CK656696	-3.346	ORESTES	Histidyl-tRNA synthetase	*M musculus*	Q61035	5E-84
EW997520	-3.337	SSH	Copine-related	*A. thaliana*	NP_564907.1	3E-70
EW997051	-3.324	SSH	Cytochrome Oxidase subunit Vb	*C. elegans*	NP_492601.1	2E-12
EW997435	-3.315	SSH	Unknown			
CO870229	-3.300	ORESTES	Unknown			
EW997131	-3.210	SSH	Tyrosyl-tRNA synthetase	*H. sapiens*	AAB39406.1	8E-26
EW997037	-3.151	SSH	Cytochrome c1 precursor	*A. thaliana*	NP_198897.1	5E-44
DY523263*^	3.502	SSH	Unknown			
EW996837*	3.710	SSH	Unknown			
DY523267*^	3.938	SSH	Unknown^4^			
EW997405	4.542	SSH	Unknown^4^			
EW996814*	5.308	SSH	Unknown			

Genes identified as differentially expressed between resistant and susceptible snail lines, ranked in order of mean (across the 4 replicate arrays) fold difference in expression, based on normalized mean intensity ratios. Negative fold difference values indicate resistant-associated clones.

^a^Source indicates whether the clone was from a suppression subtractive hybridization (SSH) library or open reading frame EST (ORESTES) library.

^b^The name, organism, accession number and e-value given for the best match with GenBANK using BLASTX.

* Chosen for qPCR

^ also confirmed from SSH experiment

^1–9 ^clusters of overlapping sequences

**Figure 3:**
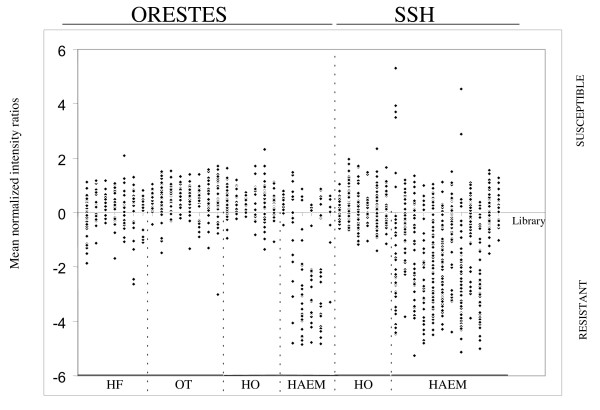
**Scatter plot of mean (across the 4 replicate arrays) fold difference in expression, based on normalized mean intensity ratios across each sequenced library, demonstrating the origin (tissue and method) of the differentially expressed genes**. ORESTES – open reading frame EST libraries, SSH – suppression subtractive hybridization libraries. OT – ovotestis, HAEM – haemocytes, HO – haemopoietic organ, HF – headfoot.

All the identified differentially expressed genes were originally sequenced from haemocyte libraries (Fig. 3); although many other transcripts were present in the samples, they were not differentially expressed. Just over a quarter (25.7%; n = 27) of the differentially expressed genes were from ORESTES and the remaining from SSH. Clustering the identified differentially expressed sequences revealed 9 clusters, one with 5 sequences and 8 with two (Table 2), leaving 98 unique sequences or clusters. One of the clusters contained two susceptible specific sequences leaving 4 unique susceptible and 94 resistant specific sequences. All of the susceptible-specific transcripts had no database matches and were derived from SSH. They included 2 sequences (DY523263 and cluster 4 – DY523267/EW997405) that had previously been identified as differentially expressed between resistant and susceptible snail haemocytes by screening macroarrays of SSH clones and confirmed with RT-PCR [27].

### Annotation of identified sequences

**Figure 4:**
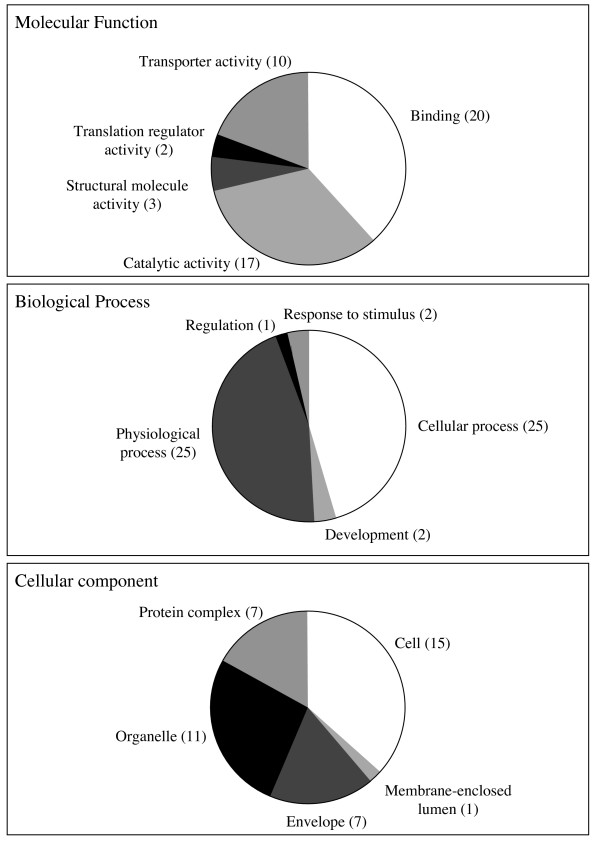
**Gene ontologies for the 29 (of 110) differentially expressed genes that had GO matches**. All those shown here are resistant specific, since the susceptible-specific genes identified no homologous genes.

Sixty of the 98 sequences or clusters did not identify other GenBank sequences in BlastX searches (Table 2) and were categorized as genes with unknown function. The functions of the other identified genes were more closely examined using gene ontology and KEGG pathway analysis. Gene ontologies were assigned for 29 genes of the 110 differentially expressed transcripts (Fig. 4), including, among other genes (see Table 2); titin (CK149228), importin 7 (EW997446), copine 1 (EW997520), elongation factor 1α (EF-1α: EW997053) and EF-2 (EW997067/EW997555 in cluster 7) and the stress response protein HSP70 (CK656737/CK656707 in cluster 3). KEGG pathway analysis identified 24 enzymes with 27 clones in a number of metabolic pathways (Table 3), including malate dehydrogenase (EW997411) involved in carbohydrate metabolism; six genes involved in oxidative phosphorylation in energy metabolism and six clones involved in amino acid metabolism including two clones homologous to ornithine decarboxylase 1 (ODC1) (EW996975 and EW996976). Although both sequences identified ODC1 in blast searches, they did not cluster at the nucleotide level, despite identifying overlapping protein sequence and are likely to be paralogous. Two genes coding for ODC1 have been found in both *Xenopus laevis *[31] and *Drosophila melanogaster *[32]. Six genes involved in translation were identified as well as the ubiquitin conjugating enzyme, UBE2D/E (CK656733), involved in folding, sorting and degradation. We also identified one sequence with a weak homology to cathepsin L cysteine protease (CO870188), involved in degradation of exogenous and endogenous proteins in lysosomes. The function of these genes and their potential involvement in the snail IDS is discussed further below.

**Table 3:** KEGG pathways identified by differentially expressed genes.

**01100 Metabolism**		
**01110 Carbohydrate Metabolism**		
00020 Citrate cycle (TCA cycle) [PATH:ko00020]		
K00026 E1.1.1.37B, mdh; malate dehydrogenase [EC:1.1.1.37]	EW997411	
00620 Pyruvate metabolism [PATH:ko00620]		
K00026 E1.1.1.37B, mdh; malate dehydrogenase [EC:1.1.1.37]	EW997411	
00630 Glyoxylate and dicarboxylate metabolism [PATH:ko00630]		
K00026 E1.1.1.37B, mdh; malate dehydrogenase [EC:1.1.1.37]	EW997411	
**01120 Energy Metabolism**		
00190 Oxidative phosphorylation [PATH:ko00190]		
K03942 NDUFV1; NADH dehydrogenase (ubiquinone) flavoprotein 1 [EC:1.6.5.3 1.6.99.3]	EW996832	
K00412 CYTB; ubiquinol-cytochrome c reductase cytochrome b subunit [EC:1.10.2.2]	EW997412	
K00413 CYT1; ubiquinol-cytochrome c reductase cytochrome c1 subunit [EC:1.10.2.2]	EW997037	
K02256 COX1; cytochrome c oxidase subunit I [EC:1.9.3.1]	EW997127	
K02265 COX5B; cytochrome c oxidase subunit Vb [EC:1.9.3.1]	EW997051	
K02137 ATPeF1O, ATP5O; F-type H+-transporting ATPase oligomycin sensitivity conferral protein [EC:3.6.3.14]	EW997569	
**01150 Amino Acid Metabolism**		
00271 Methionine metabolism [PATH:ko00271]		
K01251 E3.3.1.1, ahcY; adenosylhomocysteinase [EC:3.3.1.1]	EW996824	
00330 Arginine and proline metabolism [PATH:ko00330]		
K01881 E6.1.1.15, proS; prolyl-tRNA synthetase [EC:6.1.1.15]	CO870190	
K01581 E4.1.1.17, speF; ornithine decarboxylase [EC:4.1.1.17]	EW996975	EW996976
00340 Histidine metabolism [PATH:ko00340]		
K01892 E6.1.1.21S, hisS; histidyl-tRNA synthetase [EC:6.1.1.21]	CK656696	
00350 Tyrosine metabolism [PATH:ko00350]		
K01555 E3.7.1.2, FAH; fumarylacetoacetase [EC:3.7.1.2]	EW997017	
00400 Phenylalanine, tyrosine and tryptophan biosynthesis [PATH:ko00400]		
K01866 E6.1.1.1, tyrS; tyrosyl-tRNA synthetase [EC:6.1.1.1]	EW997131	
00220 Urea cycle and metabolism of amino groups [PATH:ko00220]		
K01581 E4.1.1.17, speF; ornithine decarboxylase [EC:4.1.1.17]	EW996975	EW996976
**01160 Metabolism of Other Amino Acids**		
00450 Selenoamino acid metabolism [PATH:ko00450]		
K01251 E3.3.1.1, ahcY; adenosylhomocysteinase [EC:3.3.1.1]	EW996824	
**01196 Xenobiotics Biodegradation and Metabolism**		
00643 Styrene degradation [PATH:ko00643]		
K01555 E3.7.1.2, FAH; fumarylacetoacetase [EC:3.7.1.2]	EW997017	
		
**01200 Genetic Information Processing**		
**01220 Translation**		
03010 Ribosome [PATH:ko03010]		
K02981 RP-S2e, RPS2; small subunit ribosomal protein S2e	EW996986	
K02977 RP-S27Ae, RPS27A; small subunit ribosomal protein S27Ae	CK656741	
00970 Aminoacyl-tRNA biosynthesis [PATH:ko00970]		
K01866 E6.1.1.1, tyrS; tyrosyl-tRNA synthetase [EC:6.1.1.1]	EW997131	
K01881 E6.1.1.15, proS; prolyl-tRNA synthetase [EC:6.1.1.15]	CO870190	
K01892 E6.1.1.21S, hisS; histidyl-tRNA synthetase [EC:6.1.1.21]	CK656696	
K04043 DNAK; molecular chaperone DnaK (Hsp70)	CK656707	CK656737
01230 Folding, Sorting and Degradation		
04120 Ubiquitin mediated proteolysis [PATH:ko04120]		
K06689 UBE2D_E, UBC4, UBC5; ubiquitin-conjugating enzyme UBE2D/E [EC:6.3.2.19]	CK656733	
		
**01300 Environmental Information Processing**		
**01310 Membrane Transport**		
02010 ABC transporters [PATH:ko02010]		
K05660 ABCB5; ATP-binding cassette, subfamily B (MDR/TAP), member 5	CK656700	
K04043 DNAK; molecular chaperone DnaK (Hsp70)	CK656707	CK656737
		
**01400 Cellular Processes**		
**01460 Immune System**		
04612 Antigen processing and presentation [PATH:ko04612]		
K01365 E3.4.22.15, CTSL; cathepsin L [EC:3.4.22.15]	CO870188	
		
**Unclassified**		
K01346 pancreatic elastase II	EW996827	
K03231 elongation factor EF-1 alpha subunit	EW997053	
K03234 elongation factor EF-2	EW997067	EW997555
K08884 serine/threonine protein kinase	EW996808	
K09054 activating transcription factor 6	EW997045	

Pathways identified by genes found only in the parasite-exposed resistant snails.

## Discussion

A *B. glabrata *cDNA microarray was successfully constructed consisting of 2053 clones from headfoot, ovotestis, haemopoeitic organ and haemoctyes of two different strains of *B. glabrata*. Screening the array with RNA from haemocytes of schistosome-exposed resistant and susceptible snail strains identified 98 differentially expressed genes or gene clusters. There was little difference between technical replicates indicating that there were no sampling effects from amplifying small amounts of starting RNA, giving confidence in the assignment of strain-associated differential expression. Examination of the expression of 9 selected genes using real-time RT-PCR on unamplified cDNA confirmed differential expression in each case.

Most of the genes identified (95.9%) were present only in the resistant snail line. That only a few differentially expressed genes were identified in susceptible snails may be due to the failure of the snail's defence system to recognize the parasite, possibly because *S. mansoni *sporocyts may mimic snail moieties (molecular mimicry) or because they may rapidly acquire molecules from the host with which to disguise themselves [33]. Alternatively, *S. mansoni *sporocysts may actively suppress a humoral response in susceptible hosts. In either case the transcripts identified in the resistant snails are genes, either constitutively over-expressed in resistant snails, not suppressed in resistant snails, or activated when the snail does recognize the parasite's presence and responds. Of the 4 genes, or gene clusters, identified in susceptible snails, two (DY523263 and cluster 4: DY523267/EW997405) had been previously identified and confirmed as differentially expressed in our earlier experiments [27] suggesting that they are consistently differentially expressed by susceptible snails. Since none has a known function it is difficult to speculate on their possible role(s).

Over half (63.8%) of the genes identified with higher expression levels in the resistant snails have no known function. Although many of these may play a significant role in defence, further characterization of their function is required to ascertain what that role might be in the snail IDS. Of the ESTs with homology to known genes, the observed differential expression of genes involved in energy metabolism, in particular oxidative phosphorylation, amino acid metabolism and genetic translation, indicates a general increase in cellular activity, consistent with generating the necessary components for mounting a defensive response. For example, EF-1α (EW997053) and EF-2 (EW997067/EW997555 in cluster 7), as well as several other proteins involved in protein synthesis, were identified, including crooked neck-like 1 protein (CK656728) involved in pre mRNA splicing. Differential expression of these types of transcripts suggests an increase in general cell activity, with increased production of new proteins in response to infection in the resistant compared to the susceptible line.

Two paralogous ornithine decarboxylase (EC 4.1.1.17) ODC1 gene fragments (EW996975 and EW996976) were identified as differentially expressed in resistant snails. ODC is the first and rate-limiting enzyme in the polyamine biosynthesis pathway, which decarboxylates L-ornithine (a product of arginase activity) to form putrescine. Polyamines (putrescine, spermidine and spermine) regulate gene expression, modulate cell signalling and are required for normal cell proliferation, important in inflammatory and infection processes [34]. Polyamines have been described as primordial stress molecules with defensive functions against diverse stresses [35], including protecting cells from DNA strand breakage induced by reactive oxygen species (ROS) and functioning directly as free radical scavengers [36,37]. Generation of ROS has been shown to play a role in sporocyst killing by molluscan haemocytes in incompatible snail-trematode systems [38,39]. Production of polyamines could protect the resistant snail's own cells from the damaging effects of ROS.

The identification of ODC in resistant snails may also imply activation of arginine metabolic pathways, which play an important role in inflammation and wound healing [40,41]. Increased levels of ODC in resistant snails indicate production of the substrate L-ornithine, inferring the depletion of L-arginine by arginase activity and subsequent inhibition of nitric oxide (NO) preventing damage to host cells. L-arginine (L-arg) is the substrate for both arginase (produces L-ornithine and urea) and nitric oxide synthase (NOS) (produces L-citrulline and NO). In inflammatory diseases, it is thought that NO production from L-arg is involved in the initial early host response creating an overall cytotoxic environment, whilst L-ornithine production from L-arg is involved in healing, promoting cell growth and proliferation [42,43]. In its role as host defender, NO regulates inflammatory responses and acts as an effector molecule of haemocyte cytotoxicity against invaders (such as parasites), whilst at the same time when produced in excessive amounts, NO is cytotoxic not only to the invading schistosome but also to the snail hosts own cells. Hence, infected snail hosts must strive to find a balance between anti-schistosome and cytotoxic effects of NO towards its own cells. NO has been shown to mediate host-protective responses in a variety of parasitic infections [44] including the killing of *S. mansoni *sporocysts by haemocytes from resistant *B. glabrata *[45]. Differential expression of ODC in resistant snails may suggest an involvement in snail defence by scavenging the damaging free radicals produced during the production of ROS by haemocytes for cytotoxic killing of sporocysts; in regulating production of cytotoxic NO by depleting the competing substrate; or, with the production of polyamines leading to DNA protection and cell proliferation, aiding wound healing following miracidial penetration.

Also identified as differentially expressed in the resistant snail line, HSP70 (CK656737/CK656707 in cluster 3) was previously identified, using differential display, as upregulated only in the resistant snails after parasite exposure [21], verifying our original suggestion that upregulation of HSP70 is an expected response to infection, and is absent from the susceptible snails. The induction of heat shock or stress proteins represents a homeostatic defence mechanism of cells to metabolic and environmental insults, and this response has previously been demonstrated in molluscs, for example in oyster haemocytes in response to environmental stimulus [46]. Experiments with mollusc haemocytes derived from *Crassostrea gigas *and *Haliotis tuberculata *demonstrated that the HSP70 gene promoter is induced by noradrenaline and α-adrenergic stimulations [47] showing that the response in these cells is integrated with neuroendocrine signalling pathways.

Several proteins potentially involved in cell signalling were also identified in the resistant snails only. One transcript (CK149228) was found to be homologous to titin (sometimes known as connectin), which is a giant springlike protein responsible for passive tension generation and for positioning of the thick filament at the centre of vertebrate striated muscle sacromeres. This protein has multiple elastic and signalling functions derived from a complex subdomain structure, including a series of immunoglobulin (Ig) domains. The kinase domain of titin initiates a signal transduction cascade that controls sarcomere assembly, protein turnover, and transcriptional control in response to mechanical changes in vertebrates [for review see [48]]. Smaller but related molecules have been identified in invertebrate striated or smooth muscles, variously named mini-titins, projectins or twitchins (in molluscs), depending on their origin [49,50]. The expression of this gene in resistant snails may indicate a signalling or muscle response to parasite infection that is not initiated in susceptible snails. In addition to titin, a cytoplasmic intermediate filament (IF) protein (CK656726) was identified only in the resistant snails. Rather than merely providing a cellular framework, recent research has demonstrated that IFs are dynamic, motile elements of the cytoskeleton in vertebrate cells [51]. An IF protein has previously been identified in *B. glabrata*, using a comparative proteomic approach, as differentially expressed in snails resistant to *E. caproni *[52] and it was suggested that this gene could be a candidate to explain differences in susceptibility/resistance, considering the major role of haemocyte mobility and adherence capabilities in defence to the parasite. Interestingly, uninfected susceptible snails demonstrated higher levels of the IF gene transcript; however post-exposure to the parasite the resistant snails showed a significant increase in transcript levels, while susceptible snails had a decrease. Our sequence does not identify this transcript either at the nucleotide or protein level and demonstrates close homology (e-value: 6.4e-25) to a neuronal IF, neurofilament protein NF70 from *Helix aspersa *[53] one of the type IV IFs. It has also been suggested that some IFs participate in signalling processes by providing a scaffold to bring together activated MAP kinases (such as extracellular signal-regulated kinase, ERK) with other molecules [54].

Other molecules potentially involved in signaling processes in the cell are importin 7 and copine 1. A gene fragment homologous to importin 7 (EW997446) was identified in the resistant snails. This protein functions as a nuclear import cofactor, and in *Drosophila*, has been implicated in the control of multiple signal transduction pathways, including the direct nuclear import of the activated (phosphorylated) form of MAP kinase (ERK) [55]. *S. mansoni *excretory secretory products (ESPs) and whole sporocysts have been shown to affect ERK signalling in the haemocytes of susceptible snails, but not resistant ones [56] suggesting that the disruption of ERK signalling in haemocytes facilitates *S. mansoni *survival within susceptible *B. glabrata*. Another gene, copine 1 (EW997520), implicated in membrane trafficking [57] and signal transduction [58] was identified in the resistant snails. Copine 1 is a calcium-dependent membrane binding protein which, in mammals, has been shown to regulate the NF kappa B transcriptional responses [59]. In *Arabidopsis thaliana*, copine 1 is suggested to play a role in plant disease resistant responses, possibly as a suppressor of defense responses including the hypersensitive cell death response [60].

We identified two hydrolytic enzymes in haemocytes from the resistant snail line, elastase (EW996827) and cathepsin L-like protease precursor (CO870188), also known as cysteine proteinase. Higher levels of cysteine proteinase activity have previously been observed in hepatopancreas extracts from resistant (BS-90) *B. glabrata *when compared to susceptible (M-line) snails [61]. Cathepsin L, cathepsin B and elastase were also identified among other hydrolytic enzymes from a hepatopancreas EST library derived from resistant snails and cathepsin B demonstrated greater up-regulation in resistant snails compared to susceptible snails upon parasite exposure [61]. In invertebrates, cysteine proteases play a major role in the lysosomal proteolytic system, responsible for intracellular protein degradation [62]. This role, in the lysis of phagocytosed particles, may be significant in the breakdown of encapsulated sporocysts or phagocytosed parasite components. In addition to a role as a scavenger for the clearance of unwanted proteins, this protease plays an important role in antigen processing and presentation in mammalian immune systems [63,64] and lysosomal proteases have been implicated in innate immunity in insect haemocytes [65]. Genome-wide analysis of immune challenged *Drosophila *revealed elevated expression of cathepsin L when infected with either Gram-positive bacteria or fungi [66]. Similarly, cathepsin L was highly expressed in WSSV (white spot syndrome virus) resistant shrimp, suggesting that it is involved in defence responses [67]. A cathepsin L-like gene (EE049537) has previously been identified in *B. glabrata *susceptible to *Echinostoma caproni *infection [68]. Our sequence (CO870188) does not cluster with this and identifies (by BLAST) a cathepsin-like domain 3' to sequence identified by the other gene fragment, suggesting that the ESTs may be two non-overlapping parts of the same gene. The discrepancy between the observed changes in gene expression may be due to the different response elicited by two different parasite species. *S. mansoni *fails to produce a response in susceptible snails as they fail to recognise the presence of the parasite, while in echinostome infections a defence response may be mounted by the snail, but is interfered with by the parasite [69].

Differential expression of ubiquitin conjugating enzyme (UBE2D/E) E2 (EC6.3.2.19) (EW997520) was detected in exposed resistant snails and may indicate removal of the phagocytosed sporocyst. Ubiquitination plays an important role in various cellular functions including apoptosis, cell cycle progression, transcription and endocytosis [70]. A major role is regulating the half-life of proteins by targeting them for 26S proteasomal degradation, removing denatured, damaged or improperly translated proteins.

In this study we compared haemocytes obtained from resistant and susceptible snail strains 2 to 24 h after exposure to *S. mansoni*, and have found differentially expressed transcripts potentially involved in a range of responses from signalling and inflammation responses through to lysis of proteinacous products (e.g. encapsulated sporocysts, or phagocytosed parasite components) and processing/degradation of such targeted products by ubiquitination. In future, examination of biological replicates will increase the confidence that the transcripts identified in this study are truly significant in the snail IDS, in addition to demonstrating the amplification techniques utilized in this study are robust. Examination of a series of narrower time slots will enable us to unravel the sequence of processes involved and highlight genes initiating the cascade in resistant (responsive) hosts. A simultaneous comparison of both parasite-exposed and unexposed snails from both snail lines will also determine if the basis of the snail's resistance is due to an underlying difference in gene expression between the strains, or from differences in their response to the parasite. Such comparisons may also be significant in determining why susceptible snails do not respond to infection, either by non-recognition of invading parasite or by active suppression of innate response by the parasite, which may be indicated if *S. mansoni*-exposure results in significantly more down-regulated genes in susceptible snails. At present we can only speculate on the function in *B. glabrata *of the genes identified. Future expression experiments involving RNAi or *in situ *hybridization may elucidate their role in resistance.

## Conclusion

In conclusion, the advent of the first cDNA microarray for *B. glabrata *leads the way for detailed analysis of the *B. glabrata *transcriptome and can be developed further to include more cDNAs as these become available. The array described here is particularly suited for analysis of snail haemocytes and their role in the snail IDS since it contains a large proportion of genes sequenced from haemocytes. Despite the current limited size of the array, prior enrichment for differentially expressed genes using the SSH approach has enabled us to identify a number of genes and pathways differentially expressed in the resistant snail line and potentially involved in the defence of resistant snails to schistosome infection. These include hydrolytic enzymes such as elastase and the cysteine protease, cathepsin L; ornithine decarboxylase, involved in the production of polyamines; HSP70; potential signalling molecules importin 7 and copine 1 and transcription enzymes such as EF-1α and EF-2. Continued development of this array has great potential for examining and understanding the functions of the *B. glabrata *transcriptome.

## Methods

### SSH library sequencing and bioinformatic analysis

8 SSH libraries were available for sequencing [27]. 192 clones (2 × 96), selected at random from each library, were picked into 0.5 ml LB and grown up overnight. 10 μl PCRs with M13 forward and reverse primers were carried out to check insert size and the presence of a single insert and, from these, 96 colonies were chosen for 100 μl PCRs. PCRs contained 1 × NH_4 _reaction buffer (Bioline, London, UK), 2.5 mM MgCl_2_, 0.2 mM dNTP, 0.2 μM each M13 Forward and Reverse primers and 0.025 U/μl PCR *Taq *polymerase (Bioline, London, UK). Cycling conditions were: 94°C for 2 min, then 35 cycles of 94°C for 30 sec, 58°C for 30 sec and 72°C for 1 min 30 sec, then 10 min at 72°C. Glycerol stocks for the selected colonies were stored at -80°C. PCR products were purified using Multiscreen PCR filter plates (Millipore, Billerica, USA) then cycle-sequenced directly using BigDye kit (Applied Biosystems, Foster City, USA) and T7 primer and run on ABI 377 or capillary sequencers. Vector, primer and poor quality sequences were removed using Sequencher 3.1.1 (GeneCodes Corp., Ann Arbor, USA.). Cluster analysis was performed in SeqTools  using BlastN score values (cutoff value 0.5) and used to calculate percentage redundancy. For each library BlastN and BlastX [71] searches were run and ribosomal, short (< 80 bp) and duplicate sequences were removed, although overlapping sequences were retained. Duplicate sequences between libraries were retained. Sequences were submitted to GenBank (EW996689-EW997658). Gene Ontology functions were assigned using GOblet . KEGG (Kyoto Encyclopaedia of Genes and Genomes) pathway analysis was carried out using the KEGG automatic annotation server (KAAS) for ortholog assignment and pathway mapping .

### Microarray construction

1062 ORESTES clones available from the first 27 ORESTES libraries [6] (GenBank numbers CK149151-CK149590, CK656591-CK656938, CO870183-CO870449, CV548035-CV548805, EG030731-EG030747) and 980 SSH clones (GenBank numbers EW996689-EW997658) were picked using a Microlab Star robotic work station (Hamilton) and transferred to 384 well plates. 11 clones from our previous differential display studies were also included (GenBank numbers CK136129, CK136132-CK136138) including a HSP70 sequence; a gene coding for a protein with globin-like domains and several genes with unknown functions which were shown to be upregulated in the resistant snail line after parasite exposure [21], as well as 3 clones containing overlapping regions of CYP320A (GenBank Accession AY922309) [72] which was identified from a 70% resistant snail line [20]. A total of 2053 cDNA clones (50–200 ng/μl) were printed in duplicate within each subarray. Controls were also included: yeast tRNA (250 ng/μl); *B. glabrata *genomic DNA, NHM3017, NHM1742 (200 ng/μl); pGem (purified vector with no insert) (75 ng/μl), two specific genes, (ribosomal 18s and cytochrome oxidase I) amplified from *S. mansoni *and blanks containing spotting buffer only. 15 μl aliquots were transferred to a second 384 well plate (Genetix) and 5 μl 4× spotting buffer (600 mM sodium phosphate; 0.04% SDS) added. The clones were printed in 16 subarrays (4 columns × 4 rows), with 18 × 17 clones in each subarray, and, since the size of the array allowed it, two arrays were printed per aminopropyl silane coated glass slide (GAPSII, Corning, at the Microarray facility at Dept of Pathology, Cambridge University), using a *Lucidea *arrayer (Amersham Biosciences). Microarrays were processed by baking for 2 h at 80°C and UV cross-linking at 600 mJ.

### Snail material and parasite exposure

60 adult *B. glabrata *snails from susceptible line (NHM Accession number 1742) and 60 from resistant line (NHM Accession number 3017, derived from BS-90 [73]) were held overnight in autoclaved snail water with 100 μg/ml ampicillin. Each snail was individually exposed to 10 *S. mansoni *miracidia (Belo Horizonte strain). Samples of 12 resistant and 12 susceptible snails were taken at 5 time periods, starting at 2, 4, 6, 8 and 24 h after exposure to the parasite. The extended sampling was designed to include all transcripts expressed over the first 24 h post parasite exposure. Snails were swiftly killed by decapitation, and the exuded haemolymph collected. Haemolymph was pooled for each sampling time and snail strain, and haemocytes pelleted by spinning at 10,000 g at 4°C for 20 min. The pellet was frozen in liquid nitrogen and stored at -80°C.

### Microarray hybridization

Total RNA was extracted from haemocytes pooled from all the time periods, using SV RNA extraction kit (Promega UK Ltd, Southampton, UK) according to the manufacturer's protocol. This kit includes DNAse treatment to eliminate genomic DNA contamination. cDNA was synthesized from 500 ng total RNA using the Smart PCR cDNA synthesis kit (BD Biosciences) according to the manufacturer's instructions and was labelled with Cy3 or Cy5 using the BioPrime DNA labelling system (Invitrogen). The labelled products were purified (Auto-seq 50 columns, Amersham), combined and precipitated. Before hybridization the microarray slides were prehybridized with hybridization buffer (40% formamide, 5× Denhardts, 5× SSC, 1 mM Sodium pyrophosphate, 1 mM Tris and 0.1% SDS) at 50°C for 1 h. The combined labelled cDNA was re-suspended in 40 μl hybridization solution, denatured at 95°C for 5 min then 50°C for 5 min, spun down, then applied to the array. Hybridizations were carried out at 50°C for 16–18 h in a humidified chamber. 3 independent SMART amplifications were made from the synthesized cDNA and 4 hybridizations were performed, including one dye swap. The slides were washed with 2 successive 5 min washes in 2× SSC at room temperature with agitation, then two in 0.2× SSC/0.1% SDS, then two in 0.1× SSC, each for 5 min and dried by spinning.

### Microarray scanning and analysis

Microarray slides were scanned sequentially for each Cy dye, at 10 μm resolution using an Axon GenePix 4100A scanner. The PMT (photo multiplying tube) was adjusted to give an average intensity ratio between channels of approximately 1. Spot finding and intensity analysis was carried out using GenePix Pro 5.0. The results were exported to Acuity 4.0. The mean pixel intensity (Feature(wavelength)-Background(wavelength)) was normalized using intensity-dependent lowess normalization for each feature [74] and consistency within each array was assessed by comparing normalized mean pixel intensity ratios (Cy3/Cy5) for each duplicate feature. Poor quality spots and low intensity data were removed. For each array the mean intensity values for pGem (vector) controls was calculated to give a background level of hybridization and only features with higher intensities than the mean plus one standard deviation threshold in either channel (Cy3 or Cy5) were retained. Consistency between array replicates was assessed by comparing mean (from the two duplicates) intensity ratios for each clone. The mean and SD of the remaining data, excluding SSH clones, were used to calculate 99% confidence limits for the normalized intensities for each array and those features which showed differential expression outside this 99% level marked. Genes that passed the 99% confidence level in 3 or 4 of the arrays were considered to demonstrate differential expression. The data from this microarray experiment has been deposited with ArrayExpress: accession E-MEXP-1710.

### Quantitative real-time PCR

Several clones were chosen for confirmation of differential expression using real-time (quantitative) PCR. Primers were designed for 9 clones and actin (Table 4) using Primer Express software (Applied Biosystems) and used for qPCR. PCRs contained Power SYBR^® ^Green Master Mix according to the manufacturers protocol (Applied Biosystems), 10 pmol of each primer and used 1 μl of 1/5 dilution of cDNA from resistant and susceptible haemocytes. PCR cycling conditions were: 50°C for 2 min, 94°C for 10 min, then 35 cycles of 94°C for 30 sec, 58°C for 30 sec and 60°C for 1 min, using the PRISM^® ^7000 Sequence Detection System (Applied Biosystems). A dissociation curve was generated in each case to check that only a single band was amplified. The results were analyzed using qGene [75]. Amplification efficiency curves [76] were generated by pooling and serially diluting the resistant and susceptible secondary PCRs generated for the SSH libraries [27] and the CT values (the cycle at which the fluorescence rises appreciably above background fluorescence) of the qPCRs were normalized to actin taking into account the amplification efficiency of each primer. Mean normalized expression (MNE') was calculated according to the equation:

**Table 4:** qPCR primers for selected candidates

Primer name	Gene name	Sequence 5'-3'	Product size (bp)	R/S^a^
DY523263F	Unknown	ACGTAGGAGACTGAGGGCACC	151	S
DY523263R		CAAATCCTCAAATATGCACGAAAC		
EW996837F	Unknown	AACCGAATCAGAGGCGACAG	143	S
EW996837R		CCGAGCATGGAATGGAAGAG		
DY523267F	Unknown	TGGTGATAAATGCTCTGGTAGCTC	117	S
DY523267R		CAGCAATATAATCAAAGGGCAATG		
EW996814F	Unknown	GTGAAGAATTGAGGATTGAACATCC	113	S
EW996814R		GAACCACCACATAGCGCAAAG		
EW996975F	ODC1	ACTTTTGATAAGGTGGGTTCTTCG	135	R
EW996975R		TGCTGAATCTATCCGACTGGC		
EW997032F	Unknown	TTAGTTGCAGGAGGAGGCTTAGC	116	R
EW997032R		CCGCTTGCACCGTATGATG		
CO870193F	Unknown	CAACTGGGTTGGGATCGTG	130	R
CO870193R		CCTGAACAATTCGGTCTCAGC		
CK656720F	Unknown	AAACTTGATGTGCGACTGATGG	96	R
CK656720R		CAAAATCATCTTCTGGGTAAAGGG		
CK149228F	Titin	GTGAATCTGAACCATGCGACTTAG	106	R
CK149228R		GCACCATCGTCAATCGTACG		
ACTINRTF	Actin	TATGTGCAAGGCAGGTTTCG	113	C
ACTINRTR		AGCTGTCCTTCTGACCCATACC		

^a ^R – resistant associated, S- susceptible associated, C- control.

$$MNE^{\prime }=\frac{\left(E_{ref}\right)\frac{CT_{ref,\text{well }1}+CT_{ref,\text{well }2}+CT_{ref,\text{well }3}}{3}}{\left(E_{target}\right)\frac{CT_{target,\text{well }1}+CT_{target,\text{well }2}+CT_{target,\text{well }3}}{3}}$$

where E_target _is the PCR amplification efficiency of the target gene; E_ref _is the PCR amplification efficiency of the reference gene; CT_target _is the threshold cycle of the PCR amplification of the target gene and CT_ref _is the threshold cycle of the PCR amplification of the reference gene [using Equation 3, see [75]]. Reactions were carried out in triplicate and a Student's t-test (2 samples, 2 tailed distribution) used to determine significant difference in expression between the two snail lines.

## Authors' contributions

AEL constructed the microarray, performed microarray experiments and analysed the microarray data, assisted by CSJ, and analysed real-time PCR data and wrote the manuscript. JS sequenced the SSH clones and assisted in the construction of the microarray. JMF and KFH helped with initial microarray analyses. RAK carried out the qPCR. DR, LRN and CSJ were involved in the experimental design and drafting of the manuscript. All authors read and approved the final manuscript.

## Supplementary Material

Additional file 1 **KEGG pathway analysis.** A comparison of KEGG pathways identified by *Biomphalaria glabrata *ESTs from ORESTES and SSH, using the non-redundant dataset of ESTs for each. Click here for file

Additional file 2 **Scatter plots comparing mean (of duplicate spots within arrays) normalized intensity ratios for each gene between replicate array hybridizations of pooled haemocyte material.** The correlation coefficients suggest a high level of correlation and a low level of variation among independent hybridizations from the technically replicated experiments. The data were previously screened to remove values below threshold levels. Clones for which data were missing in one of the compared arrays were discarded from the plot. Lines represent the line of regression (centre line) and the predicted 99% confidence intervals of the plotted data. Click here for file
