# Supplementary material for: Biomphalaria glabrata transcriptome: cDNA microarray profiling identifies resistant- and susceptible-specific gene expression in haemocytes from snail strains exposed to Schistosoma mansoni
Source: BMC Genomics. 2008 Dec 29;9:634. doi: 10.1186/1471-2164-9-634 (PMC2631019; doi:10.1186/1471-2164-9-634)
Supplement: Additional file 1 — KEGG pathway analysis. A comparison of KEGG pathways identified by Biomphalaria glabrata ESTs from ORESTES and SSH, using the non-redundant dataset of ESTs for each. [file 1471-2164-9-634-S1.doc]

##### Additional file 1- KEGG pathway analysis

A comparison of KEGG pathways identified by ESTs from ORESTES and SSH, using the non-redundant dataset of ESTs for each.

|  |  | ORESTES | ORESTES | SSH | SSH |
| --- | --- | --- | --- | --- | --- |
|  | KEGG categories represented | Enzymes | Clones | Enzymes | Clones |
| **1** | **Metabolism** |  |  |  |  |
| *1.1* | *Carbohydrate Metabolism* |  |  |  |  |
| 1.1.1 | Glycolysis / Gluconeogenesis | 4 | 5 | 2 | 3 |
| 1.1.2 | Citrate cycle (TCA cycle) |  |  | 2 | 2 |
| 1.1.3 | Pentose phosphate pathway | 2 | 3 |  |  |
| 1.1.5 | Fructose and mannose metabolism | 1 | 2 | 2 | 2 |
| 1.1.6 | Galactose metabolism | 1 | 1 |  |  |
| 1.1.7 | Ascorbate and aldarate metabolism | 2 | 2 |  |  |
| 1.1.8 | Starch and sucrose metabolism | 2 | 2 |  |  |
| 1.1.9 | Aminosugars metabolism | 1 | 1 | 1 | 1 |
| 1.1.10 | Nucleotide sugars metabolism |  |  | 1 | 1 |
| 1.1.11 | Pyruvate metabolism | 2 | 2 | 2 | 2 |
| 1.1.12 | Glyoxylate and dicarboxylate metabolism | 1 | 1 | 1 | 1 |
| 1.1.13 | Propanoate metabolism | 1 | 1 | 1 | 1 |
| 1.1.14 | Butanoate metabolism | 2 | 2 | 1 | 1 |
| 1.1.15 | C5-Branched dibasic acid metabolism |  |  | 1 | 1 |
| 1.1.17 | Inositol phosphate metabolism | 1 | 1 |  |  |
|  |  |  |  |  |  |
| *1.2* | *Energy Metabolism* |  |  |  |  |
| 1.2.1 | Oxidative phosphorylation | 6 | 6 | 27 | 76 |
| 1.2.5 | Reductive carboxylate cycle (CO2 fixation) |  |  | 2 | 2 |
| 1.2.6 | Methane metabolism | 1 | 1 |  |  |
| 1.2.7 | Nitrogen metabolism | 3 | 5 | 2 | 2 |
|  |  |  |  |  |  |
| *1.3* | *Lipid Metabolism* |  |  |  |  |
| 1.3.1 | Fatty acid biosynthesis | 1 | 1 |  |  |
| 1.3.2 | Fatty acid elongation in mitochondria |  |  | 1 | 1 |
| 1.3.3 | Fatty acid metabolism | 1 | 1 | 1 | 1 |
| 1.3.6 | Bile acid biosynthesis | 1 | 1 | 1 | 1 |
| 1.3.9 | Glycerolipid metabolism | 1 | 1 | 1 | 1 |
| 1.3.10 | Glycerophospholipid metabolism | 1 | 1 | 1 | 1 |
|  |  |  |  |  |  |
| *1.4* | *Nucleotide Metabolism* |  |  |  |  |
| 1.4.1 | Purine metabolism | 1 | 1 | 3 | 3 |
| 1.4.2 | Pyrimidine metabolism | 2 | 3 | 1 | 1 |
|  |  |  |  |  |  |
| *1.5* | *Amino Acid Metabolism* |  |  |  |  |
| 1.5.1 | Glutamate metabolism | 1 | 1 | 2 | 2 |
| 1.5.2 | Alanine and aspartate metabolism |  |  | 2 | 2 |
| 1.5.3 | Glycine, serine and threonine metabolism | 1 | 1 |  |  |
| 1.5.4 | Methionine metabolism | 1 | 1 | 1 | 1 |
| 1.5.5 | Cysteine metabolism |  |  | 3 | 3 |
| 1.5.6 | Valine, leucine and isoleucine degradation | 1 | 1 | 1 | 1 |
| 1.5.7 | Valine, leucine and isoleucine biosynthesis | 1 | 1 |  |  |
| 1.5.9 | Lysine degradation | 1 | 1 |  |  |
| 1.5.10 | Arginine and proline metabolism | 3 | 4 | 3 | 4 |
| 1.5.11 | Histidine metabolism | 2 | 3 |  |  |
| 1.5.12 | Tyrosine metabolism | 1 | 1 | 2 | 2 |
| 1.5.13 | Phenylalanine metabolism | 1 | 1 | 1 | 1 |
| 1.5.14 | Tryptophan metabolism | 2 | 2 |  |  |
| 1.5.15 | Phenylalanine, tyrosine and tryptophan biosynthesis |  |  | 4 | 5 |
| 1.5.16 | Urea cycle and metabolism of amino groups | 2 | 3 | 2 | 3 |
|  |  |  |  |  |  |
| *1.6* | *Metabolism of Other Amino Acids* |  |  |  |  |
| 1.6.1 | beta-Alanine metabolism | 2 | 3 |  |  |
| 1.6.4 | Selenoamino acid metabolism | 1 | 1 | 1 | 1 |
| 1.6.9 | Glutathione metabolism |  |  | 1 | 1 |
|  |  |  |  |  |  |
| *1.7* | *Glycan Biosynthesis and Metabolism* |  |  |  |  |
| 1.7.1 | N-Glycan biosynthesis |  |  | 2 | 2 |
|  |  |  |  |  |  |
| *1.8* | *Biosynthesis of Polyketides and Nonribosomal Peptides* |  |  |  |  |
| 1.8.6 | Polyketide sugar unit biosynthesis |  |  | 1 | 1 |
| 1.8.9 | Biosynthesis of vancomycin group antibiotics |  |  | 1 | 1 |
|  |  |  |  |  |  |
| *1.9* | *Metabolism of Cofactors and Vitamins* |  |  |  |  |
| 1.9.1 | Thiamine metabolism |  |  | 1 | 1 |
| 1.9.2 | Riboflavin metabolism |  |  | 1 | 1 |
| 1.9.4 | Nicotinate and nicotinamide metabolism |  |  | 1 | 1 |
| 1.9.5 | Pantothenate and CoA biosynthesis | 1 | 2 |  |  |
| 1.9.10 | Porphyrin and chlorophyll metabolism | 1 | 1 | 1 | 5 |
| 1.9.11 | Ubiquinone biosynthesis |  |  | 3 | 9 |
|  |  |  |  |  |  |
| *1.10* | *Biosynthesis of Secondary Metabolites* |  |  |  |  |
| 1.10.4 | Limonene and pinene degradation | 2 | 2 |  |  |
| 1.10.6 | Stilbene, coumarine and lignin biosynthesis | 1 | 1 |  |  |
| 1.10.8 | Alkaloid biosynthesis I |  |  | 1 | 1 |
| 1.10.9 | Alkaloid biosynthesis II |  |  | 1 | 1 |
| 1.10.12 | Streptomycin biosynthesis | 1 | 1 | 1 | 1 |
| 1.10.16 | Novobiocin biosynthesis |  |  | 1 | 1 |
|  |  |  |  |  |  |
| *1.11* | *Biodegradation of Xenobiotics* |  |  |  |  |
| 1.11.4 | gamma-Hexachlorocyclohexane degradation | 1 | 1 |  |  |
| 1.11.5 | 3-Chloroacrylic acid degradation |  |  |  |  |
| 1.11.6 | 1,1,1-Trichloro-2,2-bis(4-chlorophenyl)ethane (DDT) degradation |  |  |  |  |
| 1.11.7 | 2,4-Dichlorobenzoate degradation |  |  | 1 | 1 |
| 1.11.8 | 1,2-Dichloroethane degradation | 1 | 1 |  |  |
| 1.11.10 | Styrene degradation |  |  | 1 | 1 |
| 1.11.14 | Fluorene degradation | 1 | 1 |  |  |
| 1.11.17 | Benzoate degradation via hydroxylation |  |  | 1 | 1 |
| 1.11.18 | Atrazine degradation | 1 | 1 |  |  |
| 1.11.20 | 1- and 2-Methylnaphthalene degradation |  |  |  |  |

|  | | KEGG categories represented | |  | |  | |  | |  |
| --- | --- | --- | --- | --- | --- | --- | --- | --- | --- | --- |
| **2** | **Genetic Information Processing** | |  | |  | |  | |  | |
| *2.1* | *Transcription* | |  | |  | |  | |  | |
| 2.1.2 | RNA polymerase | |  | |  | | 1 | | 1 | |
| 2.1.3 | Basal transcription factors | | 1 | | 1 | |  | |  | |
|  |  | |  | |  | |  | |  | |
| *2.2* | *Translation* | |  | |  | |  | |  | |
| 2.2.2 | Ribosome | | 7 | | 7 | | 32 | | 37 | |
| 2.2.3 | Aminoacyl-tRNA biosynthesis | | 11 | | 23 | | 14 | | 15 | |
|  |  | |  | |  | |  | |  | |
| *2.3* | *Folding, Sorting and Degradation* | |  | |  | |  | |  | |
| 2.3.5 | SNARE interactions in vesicular transport | |  | |  | | 1 | | 1 | |
| 2.3.7 | Ubiquitin mediated proteolysis | | 2 | | 2 | | 1 | | 1 | |
| 2.3.8 | Proteasome | | 3 | | 4 | | 4 | | 4 | |
|  |  | |  | |  | |  | |  | |
| **3** | **Environmental Information Processing** | |  | |  | |  | |  | |
| *3.1* | *Membrane Transport* | |  | |  | |  | |  | |
| 3.1.1 | ABC transporters | | 5 | | 10 | | 4 | | 4 | |
|  |  | |  | |  | |  | |  | |
| *3.2* | *Signal Transduction* | |  | |  | |  | |  | |
| 3.2.1 | Two-component system | | 3 | | 3 | |  | |  | |
| 3.2.2 | MAPK signalling pathway | | 2 | | 2 | | 4 | | 4 | |
| 3.2.3 | Wnt signalling pathway | | 3 | | 3 | | 2 | | 2 | |
| 3.2.4 | Notch signalling pathway | | 2 | | 2 | | 2 | | 2 | |
| 3.2.6 | TGF-beta signalling pathway | | 1 | | 1 | |  | |  | |
| 3.2.7 | VEGF signalling pathway | | 2 | | 2 | | 3 | | 4 | |
| 3.2.8 | Jak-STAT signaling pathway | |  | |  | | 1 | | 1 | |
| 3.2.9 | Calcium signalling pathway | | 3 | | 5 | | 5 | | 8 | |
| 3.2.10 | Phosphatidylinositol signalling system | | 2 | | 2 | | 1 | | 4 | |
| 3.2.11 | mTOR signaling pathway | |  | |  | | 1 | | 1 | |
|  |  | |  | |  | |  | |  | |
| *3.3* | *Signalling Molecules and Interaction* | |  | |  | |  | |  | |
| 3.3.3 | ECM-receptor interaction | | 2 | | 2 | | 1 | | 1 | |
| 3.3.4 | Cell adhesion molecules (CAMs) | | 2 | | 2 | |  | |  | |
|  | |  | |  | |  | |  | |  |
| **4** | **Cellular Processes** | |  | |  | |  | |  | |
| *4.1* | *Cell Motility* | |  | |  | |  | |  | |
| 4.1.3 | Regulation of actin cytoskeleton | | 12 | | 15 | | 6 | | 8 | |
|  |  | |  | |  | |  | |  | |
| *4.2* | *Cell Growth and Death* | |  | |  | |  | |  | |
| 4.2.1 | Cell cycle | | 3 | | 3 | | 1 | | 2 | |
| 4.2.2 | Cell cycle-yeast | | 4 | | 4 | | 1 | | 1 | |
| 4.2.3 | Apoptosis | | 4 | | 8 | | 2 | | 4 | |
|  |  | |  | |  | |  | |  | |
|  |  | |  | |  | |  | |  | |
| *4.3* | *Cell Communication* | |  | |  | |  | |  | |
| 4.3.1 | Focal adhesion | | 9 | | 11 | | 5 | | 7 | |
| 4.3.2 | Adherens junction | | 4 | | 6 | | 2 | | 4 | |
| 4.3.3 | Tight junction | | 5 | | 7 | | 2 | | 4 | |
| 4.3.4 | Gap junction | | 5 | | 13 | | 2 | | 7 | |
|  |  | |  | |  | |  | |  | |
| *4.4* | *Endocrine System* | |  | |  | |  | |  | |
| 4.4.1 | Insulin signalling pathway | | 2 | | 2 | | 4 | | 7 | |
| 4.4.2 | Adipocytokine signalling pathway | | 1 | | 1 | |  | |  | |
| 4.4.3 | PPAR signaling pathway | |  | |  | | 1 | | 1 | |
| 4.4.4 | GnRH signaling pathway | | 2 | | 2 | | 1 | | 4 | |
| 4.4.5 | Progesterone-mediated oocyte maturation | | 1 | | 2 | | 1 | | 1 | |
|  |  | |  | |  | |  | |  | |
| *4.5* | *Immune System* | |  | |  | |  | |  | |
| 4.5.1 | Hematopoietic cell lineage | | 1 | | 1 | | 1 | | 1 | |
| 4.5.2 | Complement and coagulation cascades | | 2 | | 2 | |  | |  | |
| 4.5.3 | Toll-like receptor signalling pathway | | 1 | | 1 | | 1 | | 1 | |
| 4.5.4 | Natural killer cell mediated cytotoxicity | | 1 | | 1 | | 1 | | 1 | |
| 4.5.5 | Antigen processing and presentation | | 4 | | 5 | | 4 | | 5 | |
| 4.5.6 | T cell receptor signalling pathway | | 1 | | 1 | |  | |  | |
| 4.5.7 | B cell receptor signalling pathway | | 1 | | 1 | | 1 | | 1 | |
| 4.5.8 | Fc epsilon RI signaling pathway | |  | |  | | 1 | | 1 | |
| 4.5.9 | Leukocyte transendothelial migration | | 6 | | 7 | | 2 | | 4 | |
|  |  | |  | |  | |  | |  | |
| *4.6* | Nervous System | |  | |  | |  | |  | |
| 4.6.1 | Long-term potentiation | | 1 | | 1 | | 1 | | 4 | |
| 4.6.2 | Long-term depression | | 1 | | 1 | |  | |  | |
|  |  | |  | |  | |  | |  | |
| *4.7* | *Sensory System* | |  | |  | |  | |  | |
| 4.7.1 | Olfactory transduction | |  | |  | | 1 | | 4 | |
|  |  | |  | |  | |  | |  | |
| *4.8* | *Development* | |  | |  | |  | |  | |
| 4.8.1 | Dorso-ventral axis formation | |  | |  | | 1 | | 1 | |
| 4.8.2 | Axon guidance | | 4 | | 4 | | 1 | | 1 | |
|  |  | |  | |  | |  | |  | |
