# Supplementary figures and images for: Biomphalaria glabrata transcriptome: cDNA microarray profiling identifies resistant- and susceptible-specific gene expression in haemocytes from snail strains exposed to Schistosoma mansoni
Source: BMC Genomics. 2008 Dec 29;9:634. doi: 10.1186/1471-2164-9-634 (PMC2631019; doi:10.1186/1471-2164-9-634)

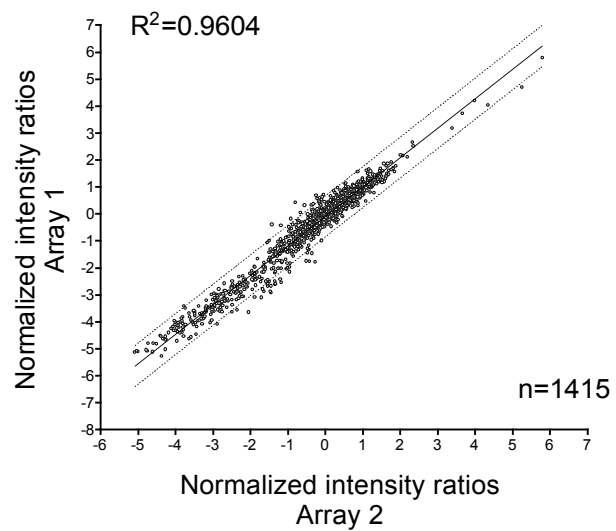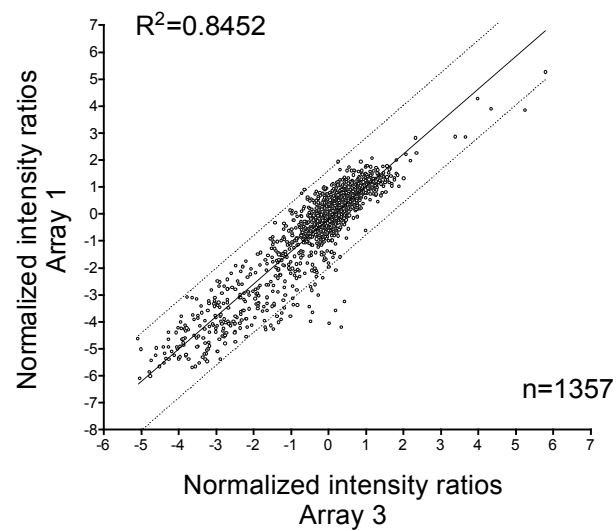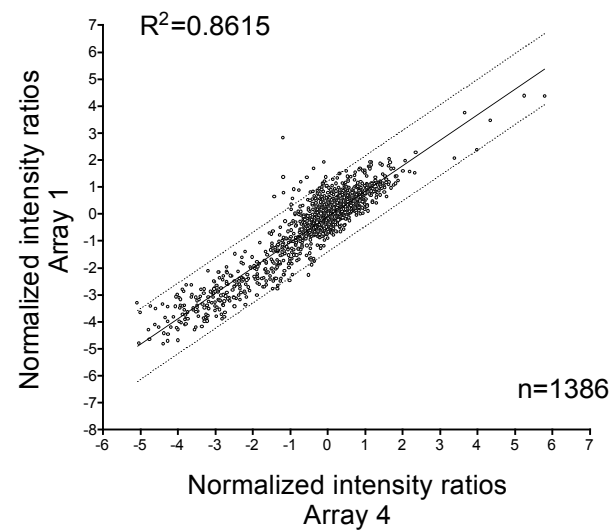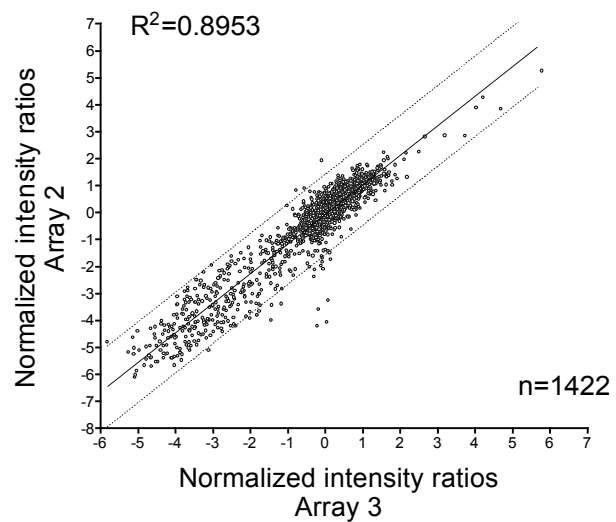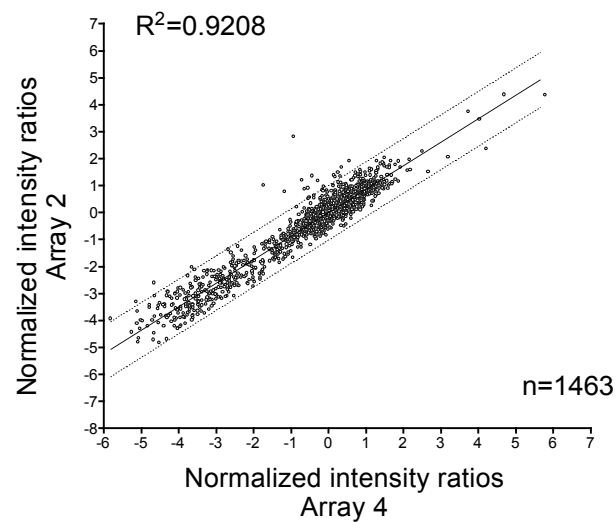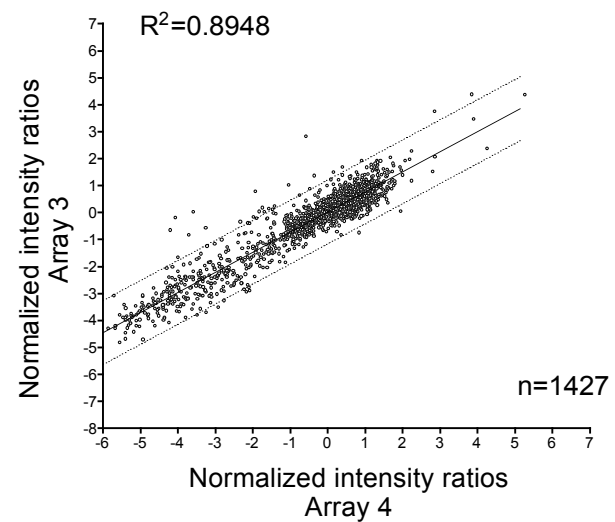

Supplement: Additional file 2 — Scatter plots comparing mean (of duplicate spots within arrays) normalized intensity ratios for each gene between replicate array hybridizations of pooled haemocyte material. The correlation coefficients suggest a high level of correlation and a low level of variation among independent hybridizations from the technically replicated experiments. The data were previously screened to remove values below threshold levels. Clones for which data were missing in one of the compared arrays were discarded from the plot. Lines represent the line of regression (centre line) and the predicted 99% confidence intervals of the plotted data. [file 1471-2164-9-634-S2.pdf]
